# HSD10 mitochondrial disease: p.Leu122Val variant, mild clinical phenotype, and founder effect in French‐Canadian patients from Quebec

**DOI:** 10.1002/mgg3.1000

**Published:** 2019-10-26

**Authors:** Paula J. Waters, Baiba Lace, Daniela Buhas, Serge Gravel, Denis Cyr, Renée‐Myriam Boucher, Geneviève Bernard, Sébastien Lévesque, Bruno Maranda

**Affiliations:** ^1^ Medical Genetics Department of Pediatrics Université de Sherbrooke‐CHUS Sherbrooke QC Canada; ^2^ CRCHUS Sherbrooke QC Canada; ^3^ Medical Genetics Department of Pediatrics CHU de Québec‐Université Laval Quebec Canada; ^4^ Medical Genetics Department of Specialized Medicine MUHC Montreal Canada; ^5^ Department of Human Genetics McGill University Montreal Canada; ^6^ Neurology Department of Pediatrics CHU de Québec‐Université Laval Quebec QC Canada; ^7^ Departments of Neurology/Neurosurgery and Pediatrics McGill University Montreal Canada; ^8^ RI‐MUHC Montreal Canada

**Keywords:** 2‐methyl‐3‐hydroxybutyryl‐CoA dehydrogenase, 17‐beta‐hydroxysteroid dehydrogenase X, HADH2, HSD10, HSD17B10, MHBD deficiency, mitochondrial disease, MRPP2, SDR5C1

## Abstract

**Background:**

HSD10 mitochondrial disease (HSD10MD), originally described as a deficiency of 2‐methyl‐3‐hydroxybutyryl‐CoA dehydrogenase (MHBD), is a rare X‐linked disorder of a moonlighting protein encoded by the *HSD17B10*. The diagnosis is usually first suspected on finding elevated isoleucine degradation metabolites in urine, reflecting decreased MHBD activity. However, it is now known that clinical disease pathogenesis reflects other independent functions of the HSD10 protein; particularly its essential role in mitochondrial transcript processing and tRNA maturation. The classical phenotype of HSD10MD in affected males is an infantile‐onset progressive neurodegenerative disorder associated with severe mitochondrial dysfunction.

**Patients, Methods, and Results:**

In four unrelated families, we identified index patients with MHBD deficiency, which implied a diagnosis of HSD10MD. Each index patient was independently investigated because of neurological or developmental concerns. All had persistent elevations of urinary 2‐methyl‐3‐hydroxybutyric acid and tiglylglycine. Analysis of *HSD17B10* identified a single missense variant, c.364C>G, p.Leu122Val, in each case. This rare variant (1/183336 alleles in gnomAD) was previously reported in one Dutch patient and was described as pathogenic. The geographic origins of our families and results of haplotype analysis together provide evidence of a founder effect for this variant in Quebec. Notably, we identified an asymptomatic hemizygous adult male in one family, while a second independent genetic disorder contributed substantially to the clinical phenotypes observed in probands from two other families.

**Conclusion:**

The phenotype associated with p.Leu122Val in *HSD17B10* currently appears to be attenuated and nonprogressive. This report widens the spectrum of phenotypic severity of HSD10MD and contributes to genotype–phenotype correlation. At present, we consider p.Leu122Val a “variant of uncertain significance.” Nonetheless, careful follow‐up of our patients remains advisable, to assess long‐term clinical course and ensure appropriate management. It will also be important to identify other potential patients in our population and to characterize their phenotype.

## Graphical abstract

HSD10 mitochondrial disease (HSD10MD) is a rare X‐linked disorder, with a classical phenotype of infantile‐onset progressive neurodegeneration associated with severe mitochondrial dysfunction. We describe patients from four unrelated families, showing an unusually mild clinical phenotype. All patients have the p.Leu122Val variant in the *HSD17B10*, reflecting a founder effect.

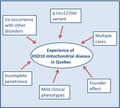

## 1 INTRODUCTION

HSD10 mitochondrial disease (HSD10MD; OMIM 300438) is a rare X‐linked metabolic disorder caused by pathogenic variants in the *HSD17B10* (17‐beta‐hydroxysteroid dehydrogenase X) gene (OMIM 300256) (Zschocke, 2012). It was originally described as a deficiency of 2‐methyl‐3‐hydroxybutyryl‐CoA dehydrogenase (MHBD; http://www.chem.qmul.ac.uk/iubmb/enzyme/EC1/1/1/178.html) (Ofman et al., 2003; Zschocke et al., 2000) recognized by increased urinary concentrations of intermediate metabolites in the isoleucine degradation pathway, 2‐methyl‐3‐hydroxybutyrate and tiglylglycine. However, HSD10 is a “moonlighting protein” enzymatically active as a dehydrogenase toward diverse substrates in vitro and demonstrating nonenzymatic binding with various other proteins (Yang, He, & Miller, 2011; Zschocke, 2012). Notably, it is an integral component (MRPP2) of the mitochondrial RNase P protein complex, crucial for structural integrity of that complex and thus essential for mitochondrial transcript processing and tRNA maturation (Deutschmann et al., 2014; Rauschenberger et al., 2010; Vilardo & Rossmanith, 2015). Disruption of this key function, in turn damaging the respiratory chain and impairing mitochondrial structure and function, is central to the pathology of HSD10MD (Chatfield et al., 2015; Deutschmann et al., 2014; Falk et al., 2016; Rauschenberger et al., 2010; Vilardo & Rossmanith, 2015). These effects are independent of all dehydrogenase activities of HSD10 (Chatfield et al., 2015; Vilardo & Rossmanith, 2015). Specifically, residual enzymatic activity of MHBD does not correlate with severity of symptoms in patients (Rauschenberger et al., 2010). This observation indicates that symptoms in patients with HSD10MD are not caused by the accumulation of isoleucine metabolites. MHBD deficiency per se is therefore now considered essentially irrelevant to the clinical disease course. However, the associated urinary markers are still often helpful in biochemical diagnosis of HSD10MD.

Seventeen pathogenic variants in *HSD17B10* have been reported, with descriptions of patients from a total of 30 families (Table S1). Sixteen variants are missense, while the other causes abnormal splicing. No “null” variants have been identified, and complete absence of HSD10 protein would probably be incompatible with embryonic survival (Zschocke, 2012). Each variant was identified in only one or two families, except for one recurrent variant, p.Arg130Cys (c.388C>T), which largely defined the “classical infantile form” of HSD10MD observed in hemizygous males (reviewed by Zschocke, 2012). The classical phenotype typically includes a period of normal development followed by progressive neurodegeneration, with loss of speech and motor skills, increasing ataxia and choreoathetosis, visual loss and epilepsy, together with severe progressive cardiomyopathy, usually leading to death in early childhood. Fulminant neonatal‐onset presentations of HSD10MD have been observed in a few male patients hemizygous for other variants (Chatfield et al., 2015; Rauschenberger et al., 2010). There have also been a few reports of attenuated phenotypes in males, with juvenile onset, minimal neurological involvement, or a nonprogressive clinical course (Akagawa et al., 2017; Olpin et al., 2002; Richardson, Berry, Garganta, & Abbott, 2017). Some females heterozygous for *HSD17B10* variants remain asymptomatic, while others show some symptoms, generally mild, particularly nonprogressive psychomotor delay or intellectual disability (Zschocke, 2012).

Here, we describe patients from four French‐Canadian families in the province of Quebec, Canada. All were found to carry the same rare variant (c.364C>G;p.Leu122Val) in *HSD17B10*, which had previously been reported in only one patient of Dutch origin (Poll‐The et al., 2004). We evaluate the clinical significance of this variant, and consider issues related to population genetics, diagnosis, management, and family counseling.

## 2 PATIENTS AND METHODS

### 2.1 Ethical compliance

This study was approved by ethics committees at CHUS (Centre hospitalier universitaire de Sherbrooke) and MUHC (McGill University Health Centre). Written informed consent, for molecular testing, inclusion in this study and publication, was obtained from patients, parents, or legal guardians, in accordance with established ethics policies at the respective university‐affiliated hospitals. Variant and phenotype information was submitted to the corresponding LOVD database at ://databases.lovd.nl/shared/variants/HSD17B10.

### 2.2 Ascertainment of patients

The index patients, from four apparently unrelated families, were each independently assessed because of clinical suspicion of possible neurological or developmental problems. Biochemical and molecular genetic tests (Data S1) were part of their diagnostic investigations. The reference sequence used for *HSD17B10* was NM_004493.2. Following diagnosis of the index patient in each family, cascade testing of other family members was initiated when clinically appropriate.

## 3 RESULTS

**Figure 1:**
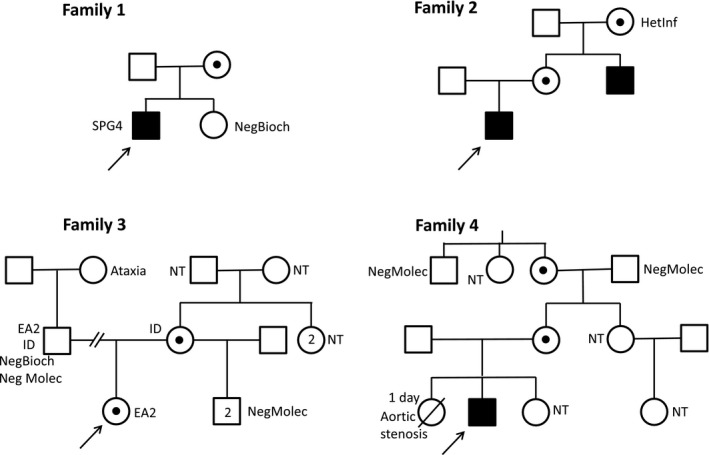
Family pedigrees, showing segregation of HSD10 mitochondrial disease. EA2, episodic ataxia type 2; HetInf, heterozygosity inferred (obligate heterozygote; no biochemical or molecular testing performed); ID, mild intellectual deficiency; NegBioch, biochemical testing (urinary organic acids and acylglycines) showed no evidence of HSD10MD; NegMolec, molecular testing indicated absence of c.364C>G (p.Leu122Val) in *HSD17B10*; NT, no biochemical or molecular testing for HSD10MD performed; SPG4, spastic paraplegia 4

Table 1 summarizes the main clinical, biochemical, and molecular findings for all identified individuals with a confirmed laboratory diagnosis of MHBD deficiency, implying a diagnosis of HSD10MD. Further context and information are provided in the family pedigrees (Figure 1) and the following text.

**Table 1:** Clinical, biochemical and molecular findings

Family	Relationship to index patient	Gender (M/F)	*HSD17B10* genotype	At time of diagnosis				Currently		Other factors
				Age	2M3HB	TG	Symptoms/signs	Age	Symptoms/signs	
1	Index patient	M	Hemiz. c.364C>G; p.Leu122Val	6 years	**94** (<18)	**74** (<5)	Spastic paraplegia Dysarthria	11 years	Spastic paraplegia Dysarthria	Pathogenic variant in *SPAST*
1	Mother	F	Heteroz. c.364C>G; p.Leu122Val	34 years	ND	ND	None	39 years	None	
2	Index patient	M	Hemiz. c.364C>G; p.Leu122Val	19 months	**285** (<26)	**147** (<5)	Episodes of dystonia/ataxia	5 years	Dystonic episodes (diminished)	
2	Mother	F	Heteroz. c.364C>G; p.Leu122Val	28 years	**71** (<8)	**24** (<3)	None	32 years	None	
2	Maternal uncle	M	Hemiz. c.364C>G; p.Leu122Val	31 years	**87** (<8)	**119** (<3)	None	34 years	None	
3	Index patient	F	Heteroz. c.364C>G; p.Leu122Val	4 months	**135** (<20)	**15** (<7)	Developmental delay Axial hypotonia	33 months	Absence of language Motor delay Episodes of ataxia Fatigue	Pathogenic variant in *CACNA1A*
3	Mother	F	Heteroz. c.364C>G; p.Leu122Val	19 years	**25** (<8)	**42** (<3)	Mild intellectual deficiency Speech difficulty	21 years	Mild intellectual deficiency Speech difficulty	
4	Index patient	M	Hemiz. c.364C>G; p.Leu122Val	8 months	**154** (<26)	**18** (<7)	None confirmed	18 months	None	
4	Mother	F	Heteroz. c.364C>G; p.Leu122Val	31 years	**74** (<8)	**48** (<3)	None	31 years	None	
4	Maternal grandmother	F	Heteroz. c.364C>G; p.Leu122Val	58 years	ND	ND	None	59 years	None	

Note

Values above reference ranges are shown in bold. The reference sequence used for *HSD17B10* is NM_004493.2.

Abbreviations: 2M3HB, urinary 2‐methyl‐3‐hydroxybutyrate, expressed in mmol/mol creatinine (age‐related reference range in parentheses); F, female; Hemiz., hemizygous; Heteroz., heterozygous; M, male; ND, not determined.TG, urinary tiglylglycine, expressed in mmol/mol creatinine (age‐related reference range in parentheses).

### 3.1 Family 1

The proband has a history of spastic quadraparesis, predominantly affecting the lower extremities, with onset at about 6 months of age. He had delayed motor milestones and has never walked independently, but started walking with a frame at age 30 months. He underwent a rhizotomy, with suboptimal results and only temporary improvement. There is remote myelination delay. He also has dysarthria. Brain MRI at age 27 months showed nonspecific abnormal signal of the bilateral dentate nuclei and posterior nuclei of the pons, having low T1 and high T2 and FLAIR signal. The MRI findings improved over time: at age 4 years, only subtly increased T2 and FLAIR signal of the bilateral dentate nuclei and bilateral medial longitudinal fasciculi were observed.

When he was 6 years old, whole exome sequencing revealed the p.Leu122Val variant in *HSD17B10*, leading to diagnosis of HSD10MD. It also identified a de novo pathogenic variant (c.1385A>G;p.Lys462Arg) in *SPAST* (RefSeq NM_014946.3; OMIM 604277), conferring a diagnosis of Spastic paraplegia 4, autosomal dominant (SPG4; OMIM 182601). He receives pharmacological treatment for spasticity and supplementation with Coenzyme Q_10_ and riboflavin. A mitochondrial disease surveillance protocol, including periodic cardiac and ophthalmological evaluation, is in place. His mother, heterozygous for p.Leu122Val, remains asymptomatic. There is no other contributory family history.

### 3.2 Family 2

The proband was diagnosed with HSD10MD at age 19 months following a transient episode of dystonia/ataxia. At the time of diagnosis, brain MRI showed normal results, as did cardiac and ophthalmological examinations. Over time, the dystonic episodes have gradually diminished in frequency and duration. Development has remained normal and there are no other clinical concerns. No specific treatment has been given. Surveillance is ongoing.

His mother is heterozygous and asymptomatic. Biochemical and molecular testing of her brother, the proband's uncle, indicated that he also has HSD10MD reflecting hemizygosity for p.Leu122Val. However, he has no known history of neurological disease or developmental delay. He reports no clinical manifestations, in response to a questionnaire, and is cognitively normal. The proband's maternal grandmother, an obligate heterozygote, is also asymptomatic.

### 3.3 Family 3

The proband is female. At 8 weeks old, suspicion of developmental delay and possible vision problems, with signs of mild intellectual deficiency in both parents, prompted referral for genetic evaluation. At age 4 months, developmental delay and mild axial hypotonia were noted. Cortical blindness was suspected, but not confirmed over subsequent months; there were no persistent vision problems. A diet restricted in isoleucine was initiated at age 12 months, but relaxed six months later, and a “mitochondrial cocktail” introduced. She started to walk at age 15 months and spoke a few words. Brain MRI at age 23 months showed normal results.

Her mother, also heterozygous for p.Leu122Val, has mild intellectual deficiency and mild dysarthria. Trials of isoleucine‐restricted diet and mitochondrial cocktail were short‐lived, by her choice. The proband's father has mild intellectual deficiency. He has ataxia, as does his mother. He was recently found to have a pathogenic variant (c.835C>T;p.Arg279Cys) in *CACNA1A* (RefSeq NM_001127222.1; OMIM 601011), and thus diagnosed with episodic ataxia, type 2 (EA2; OMIM 108500), an autosomal dominant condition. The proband has inherited this variant.

There is no other contributory family history. At age 33 months, the proband receives Coenzyme Q10, thiamine, biotin, vitamin E, selenium, and zinc supplementation. Gross and fine motor skills are delayed, and she does not speak. She has fatigue and episodes of ataxia.

### 3.4 Family 4

Genetic consultation was motivated by suspected possible episodes of absence epilepsy and observed elevations of liver transaminases in the proband, history of neonatal death of his sister, and his mother's new pregnancy. However, epilepsy was not confirmed in the proband, and a cytomegalovirus infection explained the transient hepatic abnormalities. His sister's death was attributable to a congenital cardiac malformation, severe aortic stenosis. At age 18 months, the proband is asymptomatic with normal development. Surveillance is ongoing. Brain MRI has not been performed. He receives no treatment.

His mother and maternal grandmother, both heterozygous for p.Leu122Val, are asymptomatic. His maternal great‐uncle (the only available male relative) tested negative for p.Leu122Val. His mother's pregnancy showed a female fetus on ultrasound and prenatal testing was not requested. The female infant, now 1‐month‐old, is asymptomatic, and testing was declined.

### 3.5 The p.Leu122Val variant

The patients from all four families had a single variant in *HSD17B10*, c.364C>G;p.Leu122Val. This was previously reported in one clinically affected Dutch patient (Poll‐The et al., 2004), and his mother (heterozygous and asymptomatic). MHBD activity in this patient's fibroblasts and lymphocytes, and in the p.Leu122Val protein expressed in *Escherichia coli*, was substantially decreased (Fukao et al., 2014; Ofman et al., 2003; Poll‐The et al., 2004). Fibroblasts cultured from the patient also showed signs of abnormal mitochondrial morphology, based on results of “MitoTracker” fluorescent staining (Fukao et al., 2014). The p.Leu122Val variant was described as pathogenic (Fukao et al., 2014).

It is present in only 1/183335 alleles in gnomAD (://gnomad.broadinstitute.org). As families 1–3 in our study all came from the Chaudière‐Appalaches region of Quebec, we considered the possibility of a founder effect. Subsequent haplotype analysis (Data S1) showed a shared haplotype in the patients from these families.

## 4 DISCUSSION

We have identified patients from four apparently unrelated French‐Canadian families, who were diagnosed with MHBD deficiency/HSD10MD. In each case, a single p.Leu122Val (c.364C>G) variant was identified in *HSD17B10*, for which we demonstrated evidence of a founder effect. The clinical phenotype apparently associated with the p.Leu122Val variant is mild compared to most reported cases of HSD10MD, particularly considering the asymptomatic adult male in family 2. Moreover, other independent genetic disorders clearly contributed to neurological manifestations of the probands in families 1 and 3. Several aspects of their clinical phenotypes can readily be attributed to their coexisting conditions (SPG4 and EA2, respectively). The co‐occurrence of multiple genetic disorders in families is now increasingly being recognized, reflecting a recent widening of access to clinical whole exome sequencing (Balci et al., 2017). Families 1 and 3 in our study serve as reminders to consider such possibilities, rather than automatically attributing all clinical signs and symptoms to the first disorder identified during routine metabolic investigations. An attenuated phenotype in individuals with p.Leu122Val is compatible with the reported history of the Dutch patient, who had infantile‐onset motor delay, spasticity, and minimal language development but did not show neurological deterioration (Fukao et al., 2014; Poll‐The et al., 2004). It is also possible that p.Leu122Val may have little or no clinical impact unless combined with other modifiers, genetic or environmental.

The p.Leu122Val variant almost certainly has less profound effects on mitochondrial function than the “classic” variant p.Arg130Cys. The conservative leucine to valine substitution is expected to have relatively little effect on protein secondary structure, and therefore may have only modest effects on structural integrity of the mitochondrial RNase P multiprotein complex. Notably, p.Leu122Val change did not cause significantly decreased levels of immunoreactive HSD10 protein in fibroblasts or in *E. coli* (Fukao et al., 2014; Ofman et al., 2003). In the literature, and in reports received from clinical molecular genetics laboratories, this variant has consistently been referred to as pathogenic (or “likely pathogenic”). However, while there is strong functional evidence that it does cause MHBD deficiency, the evidence linking p.Leu122Val to significantly deleterious effects on mitochondrial function is less compelling. Our patients clearly do have a biochemical disorder of isoleucine metabolism, but this in itself is no longer considered clinically important (Chatfield et al., 2015; Deutschmann et al., 2014; Falk et al., 2016; Rauschenberger et al., 2010; Vilardo & Rossmanith, 2015). It is less certain that our patients genuinely have “HSD10 *mitochondrial disease,*” in the sense of a clinically significant disease involving deficient mitochondrial respiratory chain function. Applying consensus criteria (Richards et al., 2015) to the p.Leu122Val variant, at present we consider it a “variant of uncertain significance.”

Careful follow‐up of such patients is nonetheless important, particularly to mitigate situations of stress, such as intercurrent illnesses. Vigilance will also be needed to identify other potential HSD10MD patients in our population. Several authors have highlighted challenges in biochemical diagnosis, including elevations of urinary metabolites which were only subtle or intermittent, or which could be dismissed as nonspecific in conditions of ketosis (Richardson et al., 2017). The increases in 2‐methyl‐3‐hydroxybutyrate and tiglylglycine observed in our study (Table 1 and Data S1) were generally moderate but were high enough to raise suspicion, persistent, and interpretable, taking overall profiles and nutritional status into account. Using quantitative approaches to analysis of organic acids and acylglycines (Data S1), it seems unlikely that such patients (males, at least) would be missed. However, biochemical diagnosis might be less reliable with certain other *HSD17B10* variants having minimal effect on MHBD activity but seriously affecting mitochondrial function (Chatfield et al., 2015; Vilardo & Rossmanith, 2015; Zschocke, 2012).

In summary, this report highlights the widening spectrum of phenotypic severity of HSD10MD, emphasizes the lack of correlation between decreased MHBD activity and clinical symptoms, and contributes to genotype–phenotype correlation. Our observations place the p.Leu122Val variant at the mildest end of the spectrum of clinical manifestations and suggest incomplete penetrance. Such considerations are relevant for counseling and management as, even despite the current lack of specific treatment for HSD10MD, appropriate precautions and supportive measures can be important (Zschocke, 2012).

## CONFLICT OF INTEREST

None declared.
