# Supplementary material for: HSD10 mitochondrial disease: p.Leu122Val variant, mild clinical phenotype, and founder effect in French‐Canadian patients from Quebec
Source: Mol Genet Genomic Med. 2019 Oct 26;7(12):e1000. doi: 10.1002/mgg3.1000 (PMC6900358; doi:10.1002/mgg3.1000)
Supplement: Supplementary file 1 [file MGG3-7-e1000-s001.pdf]

530 kb

	rs2480439	rs2480442	rs5978131	rs4830346	**rs1264013**	**rs1268420**	rs1264016	rs1264018	rs414960	rs266786	rs6638360	rs198508	rs144000802	rs7880223	rs6638414	rs12842443	rs10775840	rs9699111	rs9698157	rs4129175	rs9781645	rs10854983	rs9306569	rs5913850	rs5960936	rs12690009	rs4526502	rs5961058	rs6614240
Hap1:	A	C	G	T	C	C	A	C	C	T	G	T	T	A	A	C	C	G	A	T	G	C	C	A	A	T	C	A	A
Hap2:	A	C	G	T	C	C	A	C	C	T	G	T	T	A	A	C	C	G	A	C	A	T	T	G	G	T	C	G	A
Hap3:	A	C	G	T	C	C	A	C	C	T	G	T	T	A	A	C	C	G	A	C	A	T	T	G	G	T	T	G	C
Hap4:	A	A	A	T	T	T	G	T	A	G	A	C	T	A	G	C	C	A	A	T	G	C	C	A	A	T	C	A	A
Hap5:	A	C	G	T	C	C	A	C	C	T	G	T	T	A	A	C	C	G	A	C	A	T	T	G	G	T	C	G	C
Hap6:	A	A	A	G	T	T	G	T	A	G	A	C	T	A	G	C	C	A	A	T	G	C	C	A	A	T	C	A	A
Hap7:	A	C	G	T	C	C	A	C	C	T	G	T	T	A	A	T	C	G	A	C	A	T	T	G	A	T	C	A	A
Hap8:	A	A	A	T	T	T	G	T	A	G	A	C	T	A	G	C	C	A	A	T	G	C	C	A	A	T	C	G	A
Hap9:	A	A	A	T	T	T	G	T	A	G	A	C	C	A	G	C	C	A	A	T	G	C	C	A	A	T	C	G	A
Hap10:	A	A	A	T	T	T	G	T	C	T	A	T	T	G	G	C	T	G	G	T	G	C	T	G	G	G	C	G	A
Hap11:	G	C	G	T	C	C	A	C	C	T	G	T	T	A	A	C	C	A	A	T	G	C	C	A	A	T	C	A	A
Hap12:	G	A	G	T	C	C	A	C	C	T	G	T	T	A	A	C	C	A	A	T	G	C	C	A	A	T	C	A	A
Hap13:	G	C	A	T	C	C	A	C	A	G	A	C	T	A	G	C	C	A	A	T	G	C	C	A	A	T	C	A	A
Hap14:	G	A	A	T	C	C	A	C	A	G	A	C	T	A	G	C	C	A	A	T	G	C	C	A	A	T	C	A	A

Supplementary figure S1: The single haplotype shared between patients is highlighted. The SNPs present in the block of linkage disequilibrium and the different haplotypes identified in controls are indicated. The p.Leu122Val variant is located between SNPs rs1264013 and rs1268420, indicated in bold type.
