# Supplementary material for: HSD10 mitochondrial disease: p.Leu122Val variant, mild clinical phenotype, and founder effect in French‐Canadian patients from Quebec
Source: Mol Genet Genomic Med. 2019 Oct 26;7(12):e1000. doi: 10.1002/mgg3.1000 (PMC6900358; doi:10.1002/mgg3.1000)
Supplement: Supplementary file 2 [file MGG3-7-e1000-s002.docx]

**SUPPORTING INFORMATION**

**HSD10 mitochondrial disease : p.Leu122Val variant, mild clinical phenotype and founder effect in French-Canadian patients from Quebec**

Paula J. Waters^1^, Baiba Lace^2^, Daniela Buhas^3^, Serge Gravel^1^, Denis Cyr^1^,
Renée-Myriam Boucher^4^, Geneviève Bernard^3,5-7^, Sébastien Lévesque^1^, Bruno Maranda^1^

^1^Medical Genetics Service, Dept. Pediatrics, Université de Sherbrooke-CHUS, and CRCHUS (Centre de recherche du Centre hospitalier universitaire de Sherbrooke), Sherbrooke, QC, Canada
^2^Medical Genetics Service, Dept. Pediatrics, CHU de Québec-Université Laval, Quebec, Canada
^3^Medical Genetics Division, Dept. Specialized Medicine, McGill University Health Centre (MUHC),
and Dept. Human Genetics, McGill University, Montreal, QC, Canada
^4^Neurology Service, Dept. Pediatrics, CHU de Québec-Université Laval, Quebec, Canada
^5^Dept. Neurology and Neurosurgery and Dept. Pediatrics, McGill University, Montreal, QC, Canada
^6^Child Health and Human Development Program, Research Institute of the McGill University Health Centre, Montreal, QC, Canada
^7^MyeliNeuroGene Laboratory, Research Institute of the MUHC, Montreal, QC, Canada

**Supplementary Table S1**  Reported families with pathogenic variants in the *HSD17B10* gene

| Sequence variant  cDNA Protein | | Number of families | Reference |  | Comments |
| --- | --- | --- | --- | --- | --- |
| c.34G>C | p.Val12Leu | 1 | Oerum et al., 2017 |  |  |
| c.194T>C | p.Val65Ala | 1 1 | Seaver et al., 2011 Richardson et al., 2017 |  |  |
| c.257A>G | p.Asp86Gly | 1 | Rauschenberger et al., 2010 |  |  |
| c.364C>G | p.Leu122Val | 1 | Poll-The et al., 2004 Fukao et al., 2014 |  | Personal communication to Fukao from Poll-The |
|  |  | 4 | This study |  |  |
| c.388C>T | p.Arg130Cys | 1 2 1 1 1 1 1 2 | Zschocke et al., 2000 Ensenauer et al., 2002 Sutton et al., 2003 Sass et al., 2004 Perez-Cerda et al., 2005 Cazorla et al., 2007 Garcia-Villoria et al., 2009 Zschocke, 2012 | (Ofman et al., 2003) (Ofman et al., 2003) (Yang et al., 2009) (Zschocke, 2012) | Recurrent variant, with multiple *de novo* occurrences; mechanism discussed in Zschocke, 2012 and Yang et al., 2013 |
| c.460G>A | p.Ala154Thr | 1 | Fukao et al., 2014 |  |  |
| c.470C>T | p.Ala157Val | 1 | Akagawa et al., 2017 |  |  |
| c.473C>T | p.Ala158Val | 1 | Lorea et al., 2015 (Abstract) |  |  |
| c.495A>C | p.Gln165His | 1 | Rauschenberger et al., 2010 |  |  |
| c.525A>G | p.Ile175Met | 1 | Su et al., 2017 |  |  |
| c.526G>A | p.Val176Met | 1 | Oerum et al., 2017 |  |  |
| c.574C>A | p.Arg192Arg (synonymous) | 1 | Reyniers et al., 1999 | (Lenski et al., 2007) | Variant causing abnormal splicing |
| c.628C>T | p.Pro210Ser | 2 | Garcia-Villoria et al., 2009 |  |  |
| c.634A>G | p.Lys212Glu | 1 | Falk et al., 2016 |  |  |
| c.677G>A | p.Arg226Gln | 1 1 | Garcia-Villoria et al., 2009 Su et al., 2017 |  |  |
| c.740A>G | p.Asn247Ser | 1 1 | Perez-Cerda et al., 2005 Chatfield et al., 2015 |  |  |
| c.745G>C | p.Glu249Gln | 1 | Olpin et al., 2002 | (Yang et al., 2009) |  |

The reference sequence used for HSD17B10 is NM_004493.2 (LRG_450).
Listed references provide the primary clinical descriptions of the index patients; some also include descriptions of additional family members. When not included in the case report, the sequence variant was specified in a separate reference; indicated in parentheses.

**SUPPLEMENTARY METHODS AND RESULTS**

**Biochemical diagnostic analyses**

Analysis of urinary organic acid profiles was performed by widely-used methodology, including trimethylsilyl derivatization followed by gas chromatography-mass spectrometry (GC-MS). 2-methyl-3-hydroxybutyrate was among the acids for which calibration with a standard of that molecule was included in the method. The chromatograms were also scrutinised for 2-methylacetoacetate, as this acid will often be elevated in β-ketothiolase deficiency but is not elevated in HSD10MD and therefore contributes to differential diagnosis between these two conditions (Zschocke et al., 2000; Zschocke, 2012).

Analysis of urinary acylglycine profiles was performed using a fully-quantitative liquid chromatography-tandem mass spectrometry (LC-MS/MS) method, including calibration against an isotopically-labelled internal standard for tiglylglycine (Bherer et al., 2015). This was applied as a reflex test in the diagnostic process, to support interpretation of results from urinary organic acid analysis. These tests were performed in a single clinical biochemical genetics reference laboratory, at CHUS (Centre hospitalier universitaire de Sherbrooke).

**Molecular diagnostic analyses**

The index patient of Family 1 was investigated by whole exome sequencing, with subsequent confirmation of identified variants by Sanger sequencing, followed by biochemical confirmation of HSD10 mitochondrial disease (HSD10MD).

For the index patients of families 2, 3 and 4, HSD10MD was suspected based on the observed biochemical profiles. Sanger sequencing of *HSD17B10* was then performed, in clinical molecular genetics laboratories, for confirmation of diagnosis.

For other family members, targeted Sanger sequencing was used to confirm or rule out presence of the variant previously identified in the index patient.

**Haplotype analysis**

DNA specimens from three hemizygous males (index patients from families 1 and 2, maternal uncle from family 2), and from three heterozygous females (mother from family 2, index patient and mother from family 3), were analysed alongside control samples from 14 French-Canadian male individuals. A Global Screening SNP Array (version 2, Illumina) was used (laboratory analysis of anonymized samples performed by the McGill University and Genome Quebec Innovation Centre).

The structure of linkage disequilibrium in the region around HSD17B10 was then determined using Haploview ([http://www.broad.mit.edu/mpg/haploview/) (Barrett, Fry, Maller & Daly, 2005)](http://www.broad.mit.edu/mpg/haploview/)%20(Barrett,%20Fry,%20Maller%20&%20Daly,%202005)). Haplotype blocks were defined based on D' estimates using the option “Solid Spine of LD”. The three hemizygous males were found to share a common haplotype (Supplementary figure S1; Hap 1). Results for the three heterozygous females were consistent with presence of the same haplotype. In the 14 male controls, 13 other haplotypes were identified. No control subject had Haplotype 1.

Family 4 was identified after the analysis of families 1-3 had been performed.

**Further biochemical characterisation**

After diagnosis, repeat urinary organic acid and acylglycine analyses were performed periodically during the follow-up of each of the four index patients. Apart from the characteristic elevations of 2-methyl-3-hydroxybutyrate and tiglylglycine, the profiles were otherwise unremarkable, at diagnosis and also throughout follow-up. These two metabolites remained persistently elevated above their age-related reference ranges in all specimens from all patients, with the exception of a temporary near-normalisation of values for the female index patient of family 3 during a period of dietary isoleucine/protein restriction.

Serum acylcarnitine profiles were also analysed (by widely-used methodology; tandem mass spectrometry following butylation) at least once for each of the four index patients, at diagnosis and/or during long-term follow-up (Supplementary Table S2). In each of the three hemizygous male index patients, mild elevation of C5:1, presumably reflecting tiglylcarnitine, was observed. C5-OH was never found to be elevated in any of them. For two heterozygous females, analysis of serum acylcarnitines was performed once, giving normal results. These findings overall were concordant with most other published reports, suggesting that acylcarnitine profile analysis is of somewhat limited utility in diagnosis of HSD10MD (Zschocke, 2012).

**ADDITIONAL ACKNOWLEDGEMENTS**

We wish to thank the analytical and technical staff of the Biochemical Genetics Laboratory at CHUS for their contributions to the diagnostic investigations and follow-up testing for the patients and their family members. The authors also wish to acknowledge the services provided by the McGill University and Genome Quebec Innovation Centre.

Diagnosis of Family 1 via whole-exome sequencing was made possible by Dr Bernard’s research program for which she gratefully acknowledges multiple sources of support, including grants from the Fondation du Grand Défi Pierre Lavoie, Fondation Lueur d’espoir pour Ayden, Fondation le Tout pour Loo and Réseau de Médecine Génétique Appliquée of the Fonds de Recherche en Santé du Québec, as well as support provided by Compute Canada ([www.computecanada.ca](http://www.computecanada.ca)). Dr Bernard has received the New Investigator Salary Award from the Canadian Institutes of Health Research (2017-2022).

The HSD10MD study was presented in preliminary form by Dr Waters at the 2018 meetings of the Garrod Association and of the Association des Médecins Généticiens du Québec.

**Supplementary Table S2** Serum acylcarnitine results

|  |  |  |  | **Serum acylcarnitines _____________________________________________** | | | |
| --- | --- | --- | --- | --- | --- | --- | --- |
| **Family** | **Relationship to  index patient** | **Gender  (M/F)** | ***HSD17B10*  genotype** | **Age at testing** | **C5-OH (µmol/L;  ref. ≤ 0.04)** | **C5:1 (µmol/L; ref. ≤ 0.02)** | **Rest of profile** |
| 1 | Index patient | M | Hemiz. c.364C>G; p.Leu122Val | 7y 9y | 0.01 0.03 | **0.04**  **0.05** | Normal Normal |
| 1 | Mother | F | Heteroz. c.364C>G; p.Leu122Val | NA | ND | ND | ND |
| 2 | Index patient | M | Hemiz. c.364C>G; p.Leu122Val | 19m 20m 30m 43m | 0.03  0.03 0.03 0.03 | **0.05 0.08 0.07 0.06** | Normal Normal Normal Normal |
| 2 | Mother | F | Heteroz. c.364C>G; p.Leu122Val | 28y | 0.01 | 0.02 | Normal |
| 2 | Maternal uncle | M | Hemiz. c.364C>G; p.Leu122Val | NA | ND | ND | ND |
| 3 | Index patient | F | Heteroz. c.364C>G; p.Leu122Val | 6m | 0.02 | 0.02 | Normal |
| 3 | Mother | F | Heteroz. c.364C>G; p.Leu122Val | NA | ND | ND | ND |
| 4 | Index patient | M | Hemiz. c.364C>G; p.Leu122Val | 8m | 0.04 | **0.06** | Mild elevations of several other acylcarnitines; non-specific pattern, probably secondary to nutritional status. |
| 4 | Mother | F | Heteroz. c.364C>G; p.Leu122Val | NA | ND | ND | ND |
| 4 | Maternal grandmother | F | Heteroz. c.364C>G; p.Leu122Val | NA | ND | ND | ND |

Abbreviations:
M, male; F, female; Hemiz., hemizygous; Heteroz., heterozygous; y, years; m, months;
C5-OH, C5-hydroxy acylcarnitine, representing the sum of 2-methyl-3-hydroxybutyrylcarnitine and 3-hydroxyisovalerylcarnitine;
C5:1, C5:1 acylcarnitine, representing the sum of tiglylcarnitine and 3-methylcrotonylcarnitine;
ref., reference range; NA, not applicable (acylcarnitine testing not performed); ND, not determined.
Values above reference range are shown in bold.

**REFERENCES for Supporting Information**
Akagawa, S., Fukao, T., Akagawa, Y., Sasai, H., Kohdera, U., Kino, M., … Kaneko, K. (2017). Japanese male siblings with 2-methyl-3-hydroxybutyryl-CoA dehydrogenase deficiency (HSD10 disease) without neurological regression. *JIMD Reports, 32*, 81-85. https://doi.org/10.1007/8904_2016_570

Barrett, J.C., Fry, B., Maller, J., Daly, M.J. (2005). Haploview: analysis and visualization of LD and haplotype maps. *Bioinformatics, 21*(2), 263-265. https://doi.org/10.1093/bioinformatics/bth457

Bherer, P., Cyr, D., Buhas, D., Al-Hertani, W., Maranda, B., & Waters, P.J. (2015). Acylglycine profiling: a new liquid chromatography-tandem mass spectrometry (LC-MS/MS) method, applied to disorders of organic acid, fatty acid and ketone metabolism. *Journal of Inherited Metabolic Disease, 38*(Suppl. 1), S69-S70. Abstract P-028. https://doi.org/10.1007/s10545-015-9877-x

Cazorla, M.R., Verdu, A., Perez-Cerda, C., & Ribes, A. (2007). Neuroimage findings in 2-methyl-3-hydroxybutyryl-CoA dehydrogenase deficiency. *Pediatric Neurology, 36*(4), 264-267. https://doi.org/10.1016/j.pediatrneurol.2006.11.014

Chatfield, K.C., Coughlin, C.R. II, Friederich, M.W., Gallagher, R.C., Hesselberth, J.R., Lovell, M.A., … Van Hove, J.L.K. (2015). Mitochondrial energy failure in HSD10 disease is due to defective mtDNA transcript processing. *Mitochondrion, 21*:1-10. https://doi.org/10.1016/j.mito.2014.12.005

Ensenauer, R., Niederhoff, H., Ruiter, J.P., Wanders, R.J., Schwab, K.O., Brandis, M. & Lehnert, W. (2002). Clinical variability in 3-hydroxy-2-methylbutyryl-CoA dehydrogenase deficiency. *Annals of Neurology, 51*(5), 656-659. https://doi.org/10.1002/ana.10169

Falk, M.-J., Gai, X., Shigematsu, M., Vilardo, E., Takase, R., McCormick, E., … Hou, Y.-M. (2016). A novel *HSD17B10* mutation impairing the activities of the mitochondrial RNase P complex causes X-linked intractable epilepsy and neurodevelopmental regression. *RNA Biology, 13*(5), 477-485. https://doi.org/10.1080/15476286.2016.1159381

Fukao, T., Akiba, K., Goto, M., Kuwqayama, N., Morita, M., Hori, T., … Hasegawa, Y. (2014) The first case in Asia of 2-methyl-3-hydroxybutyryl-CoA dehydrogenase deficiency (HSD10 disease) with atypical presentation. *Journal of Human Genetics, 59*(11), 609-614. https://doi.org/10.1038/jhg.2014.79

Garcia-Villoria, J., Navarro-Sastre, A., Fons, C., Perez-Cerda, C., Baldellou, A., Fuentes-Castello, M.A., … Ribes, A. (2009). Study of patients and carriers with 2-methyl-3-hydroxybutyryl-CoA dehydrogenase (MHBD) deficiency : Difficulties in the diagnosis. *Clinical Biochemistry, 42*(1-2), 27-33.
https://doi.org/10.1016/j.clinbiochem.2008.10.006

Lenski C, Kooy RF, Reyniers E, Loessner, D., Wanders, R.J., Winnepenninckx, B., … Ramser, J. (2007). The reduced expression of the HADH2 protein causes X-linked mental retardation, choreoathetosis and abnormal behavior. *American Journal of Human Genetics, 80*(2), 372-377. https://doi.org/10.1086/511527

Lorea, C.F., Sitta, A., Yamamoto, R., Paulo, P.A.F., Vairo, F.P., Rieder, C.R.M., … Saute, J.A. (2015). Case report: atypical juvenile parkinsonism and basal ganglia calcifications due to HSD10 disease. *Journal of Inherited Metabolic Disease, 38*(Suppl. 1), S210. Abstract P-384. https://doi.org/10.1007/s10545-015-9877-x

Oerum, S., Roovers, M., Leichsenring, M., Acquaviva-Bourdain, C., Beermann, F., Gemperle-Britschgi, C., … Yue, W.W. (2017). Novel patient missense mutations in the HSD17B10 gene affect dehydrogenase and mitochondrial tRNA modification functions of the encoded protein. *Biochimica et Biophysica Acta Molecular Basis of Disease, 1863*(12), 3294-3302. https://doi.org/10.1016/jbbadis.2017.09.002

Ofman, R., Ruiter, J.P.N., Feenstra, M., Duran, M., Poll-The, B.T., Zschocke, J., … Wanders, R.J.A. (2003). 2-methyl-3-hydroxybutyryl-CoA dehydrogenase deficiency is caused by mutations in the *HADH2* gene. *American Journal of Human Genetics, 72*(5),1300-1307. https://doi.org/10.1086/375116

Olpin, S.E., Pollitt, R.J., McMenamin, J., Manning, N.J., Besley, G., Ruiter, J.P.N., & Wanders, R.J.A. (2002) 2-methyl-3-hydroxybutyryl-CoA dehydrogenase deficiency in a 23-year-old man. *Journal of Inherited Metabolic Disease, 25*(6), 477-482. https://doi.org/10.1023/A:1021251202287

Perez-Cerda, C., Garcia-Villoria, J., Ofman, R., Sala, P.R., Merinero, B., Ramos, J., … Ugarte, M. (2005). 2-methyl-3-hydroxybutyryl-CoA dehydrogenase (MHBD) deficiency : an X-linked inborn error of isoleucine metabolism that may mimic a mitochondrial disease. *Pediatric Research, 58*(3), 488-491. https://doi.org/10.1203/01.pdr.0000176916.94328.cd

Poll-The, B.T., Wanders, R.J.A., Ruiter, J.P.N, Ofman, R., Majoie, C.B.L.M., Barth, P.G., & Duran, M. (2004). Spastic diplegia and periventricular white matter abnormalities in 2-methyl-3-hydroxybutyryl-CoA dehydrogenase deficiency, a defect of isoleucine metabolism: differential diagnosis with hypoxic-ischemic brain diseases. *Molecular Genetics and Metabolism, 81*(4), 295-299. https://doi.org/10.1016/j.ymgme.2003.11.013

Rauschenberger, K., Schöler, K., Sass, J.O., Sauer, S., Djuric, Z., Rumig, C., … Zschocke, J. (2010). A non-enzymatic function of 17β-hydroxysteroid dehydrogenase type 10 is required for mitochondrial integrity and cell survival. *EMBO Molecular Medicine, 2*(2), 51-62. https://doi.org/10.1002/emmm.200900055

Reyniers, E., Van Bogaert, P., Peeters, N., Vits, L., Pauly, F., Fransen, E., … Kooy, R.F. (1999). A new neurological syndrome with mental retardation, choreoathetosis, and abnormal behaviour maps to chromosome Xp11. *American Journal of Human Genetics, 65*(5), 1406-1412.
<https://doi.org/10.1086/302638>

Richardson A., Berry, G.T., Garganta, C., & Abbott, M.-A. (2017) Hydroxysteroid 17-Beta dehydrogenase type 10 disease in siblings. *JIMD Reports, 32*, 25-32.
https://doi.org/10.1007/8904_2016_547

Sass, J.O., Forstner, R., & Sperl, W. (2004). 2-methyl-3-hydroxybutyryl-CoA dehydrogenase deficiency: impaired catabolism of isoleucine presenting as neurodegenerative disease. *Brain and Development, 26*(1), 12-14. https://doi.org/10.1016/s0387-7604(03)00071-8

Seaver, L.H., He, X.-Y., Abe, K., Cowan, T., Enns, G.M., Sweetman, L., … Yang, S.-Y. (2011). A novel mutation in the *HSD17B10* gene of a 10-year-old boy with refractory epilepsy, choreoathetosis and learning disability. *Public Library of Science ONE, 6*(11):e27348. https://doi.org/10.1371/journal.pone.0027348

Su, L., Li, X., Lin, R., Sheng, H., Feng, Z., & Liu, L. (2017). Clinical and molecular analysis of 6 Chinese patients with isoleucine metabolism defects: identification of 3 novel mutations in the HSD17B10 and ACAT1 gene. *Metabolic Brain Disease, 32*(6), 2063-2071.
https://doi.org/10.1007/s11011-017-0097-y

Sutton, V.R., O’Brien, W.E., Clark, G.D., Kim, J., & Wanders, R.J. (2003). 3-hydroxy-2-methylbutyryl-CoA dehydrogenase deficiency. *Journal of Inherited Metabolic Disease, 26*(1), 69-71. https://doi.org/10.1023/a:1024083715568

Yang, S.-Y., He, X.-Y., Olpin, S.E., Sutton, V.R., McMenamin, J., Philipp, M., … Malik, M. (2009). Mental retardation linked to mutations in the HSD17B10 gene interfering with neurosteroid and isoleucine metabolism. *Proceedings of the National Academy of Science USA, 106*(35):14820-14824. https://doi.org/10.1073/pnas.0902377106

Yang, S.-Y., Dobkin, C., He, X.-Y., Philipp, M., & Brown, W.T. (2013). A 5-methylcytosine hotspot responsible for the prevalent *HSD17B10* mutation. *Gene, 515*(2), 380-384. https://doi.org/10.1016/j.gene.2012.12.064

Zschocke, J. (2012). HSD10 disease: clinical consequences of mutations in the *HSD17B10* gene. *Journal of Inherited Metabolic Disease, 35*(1), 81-89. https://doi.org/10.1007/s10545-011-9415-4

Zschocke, J., Ruiter, J.P.N., Brand. J., Lindner, M., Hoffman, G.F., Wanders, R.J.A., & Mayatepek, E. (2000). Progressive infantile neurodegeneration caused by 2-methyl-3-hydroxybutyryl-CoA dehydrogenase deficiency: a novel inborn error of branched-chain fatty acid and isoleucine metabolism. *Pediatric Research, 48*(6), 852-855. https://doi.org/10.1203/00006450-200012000-00025
